# Adolescent psychiatric hospitalization: a naturalistic observational study of statistical and clinical outcomes and moderating factors

**DOI:** 10.1186/s13034-025-00972-8

**Published:** 2025-10-28

**Authors:** Laura Nigro, Alberto Forte, Gregory Mantzouranis, Swen Courosse, Carole Kapp, Maude Schneider, Kerstin Jessica Plessen, Marco Armando, Sébastien Urben

**Affiliations:** 1 Division of Child and Adolescent Psychiatry, Department of Psychiatry, Lausanne University Hospital (CHUV), Av. Echallens 9, Lausanne, 1004 Switzerland; 2 Faculty of Biology and Medicine, University of Lausanne, Lausanne, Switzerland; 3 The FAmily and DevelOpment research center (FADO), Faculty of Social and Political Sciences, University of Lausanne, Lausanne, Switzerland; 4 Clinical Psychology Unit for Intellectual and Developmental Disabilities, Faculty of Psychology and Educational Sciences, University of Geneva, Geneva, Switzerland

**Keywords:** Reliable change index, Clinical outcomes, Adolescents, Risk factors, Hospitalization

## Abstract

**Background:**

Adolescents’ mental health issues may require psychiatric hospitalization, highlighting the need for effective interventions during hospitalization. This study explored statistical and clinical (through reliable change index; RCI) changes in mental health difficulties across hospitalization and their moderating factors potentially affecting clinical progress and discharge outcomes.

**Methods:**

We examined retrospective socio-demographic and clinical data from 593 adolescents first hospitalized at the Adolescent Psychiatric Hospitalization Unit (UHPA) of the Lausanne University Hospital (CHUV) between 2014 and 2021. Statistical significance (analyzed via t-tests for paired samples) used scores on the Health of the Nation Outcome Scales for Children (HoNOSCA) at admission and discharge, whereas RCI was used to assess clinical significance. Moderating factors were examined through multivariate regression analyses on clinical changes and outcomes at discharge.

**Results:**

Statistically, we observed a significant reduction in mental health issues, as reflected by decreases in the *“Total”*, *“Behavioral”* and *“Symptoms”* HoNOSCA scores from admission to discharge. However, clinical improvements (RCI > 1.96) were reached in 20% of patients, while 65% remained stable. Finally, considering moderating factors, while clinical changes during hospitalization remained largely unexplained, moderators such as sex, behavioral and emotional disorders (F9x), antipsychotic intake, voluntary admission, pre-hospitalization occupation, patient-clinician discharge agreement, and length of stay accounted for a significant proportion of clinical outcomes at discharge.

**Conclusions:**

While hospitalization may contribute to stabilizing acute crises, clinically meaningful improvements were limited for many patients. This highlights the importance of integrative and coordinated approaches tailored to adolescents’ heterogeneous profiles.

**Supplementary Information:**

The online version contains supplementary material available at 10.1186/s13034-025-00972-8.

## Introduction

### Adolescence and mental health

Adolescence is a critical life period associated with the onset of most mental health problems [1–3]. Indeed, three-quarters of mental disorders emerge before the age of 18 [4–6], and the prevalence of suicidal thoughts and behaviors increases during this period [7]. In fact, in 2022, over 14.5% of Swiss youth aged 15–24 reported suicidal thoughts, and suicide remains the first leading cause of death among young people, affecting 11 per 100,000 inhabitants [8]. Nonetheless, with appropriate care provided, adolescence also represents a window of opportunity for effective interventions to improve clinical and functional outcomes [9, 10]. Following a stepped-care model, interventions are tailored to the disorder’s severity [11–14], with hospitalization reserved as a last resort to manage acute, severe phases of mental illness - especially when earlier treatments prove inadequate [15] (see Table 1).

**Table 1:** Stepped care model and related intervention

Step	Symptoms	Functioning	Type of intervention	Description
Mild Symptoms	Mild distress, transient difficulties	Maintains daily activities, social/work functioning mostly intact	Psychoeducation & Self-Help Strategies	Providing information, online resources, and self-management techniques.
Moderate Symptoms	Persistent distress, moderate symptom burden	Some difficulties in school, work, or social life	Outpatient Therapy (CBT, MBT, etc.)	Individual or group therapy to address distress and prevent worsening.
Moderate to Severe Symptoms	Significant symptom burden, increased distress	Marked impairment in social, academic, or occupational functioning	Intensive Outpatient or Day Programs	Structured, frequent interventions while maintaining community support.
Severe Symptoms	Acute symptoms (e.g., psychotic symptoms, suicidality)	Severe impairment, inability to function in daily life	Crisis Intervention & Short-Term Hospitalization	Managing acute episodes, ensuring safety, and stabilizing symptoms.
Extreme Severity	Chronic, treatment-resistant symptoms	Loss of autonomy, full dependence on external care	Long-Term Hospitalization	Used when other interventions are insufficient, focusing on stabilization and long-term treatment planning.

*CBT* Cognitive Behavioral Therapy ,* MBT* Mentalization-Based Therapy

Adolescents may be hospitalized because of acute symptoms such as self-harm, suicidal ideations or behaviors, aggressive conduct, eating disorders, and depressive and/or psychotic symptoms [16, 17]. Psychiatric hospitalization for children and adolescents may be beneficial in special situations, especially when certain treatment aspects are fulfilled (e.g., a strong therapeutic alliance and specialized treatment program), resulting in symptom stabilization and crisis containment within a short period of time [18–22]. Nevertheless, despite these short-term benefits, psychiatric hospitalization has been criticized for its limited long-term effectiveness and its demanding nature, characterized by reduced autonomy, close monitoring, stigmatization, and a significant burden on families [19, 23–25].

Moreover, most studies rely on broad, global assessments of difficulties, potentially overlooking improvements in specific areas [26–28]. A more nuanced evaluation of the different dimensions of mental disorders is therefore essential—not only to obtain a finer-grained understanding of clinical outcomes but also to guide the development of targeted interventions that maximize hospitalization benefits while mitigating their adverse personal impacts [29].

### Moving forward: from statistical to clinical significance

Most studies on the effects of psychiatric hospitalization focus on identifying statistically significant changes in the severity of difficulties [29]. While such an approach effectively detects group-level differences, it does not provide insight into whether these changes are meaningful for individual patients’ meaning or whether the level of change is actually experienced by the adolescent. In other words, a statistically significant improvement across a group does not necessarily reflect a perceptible or clinically relevant change for a given patient, a distinction that is critical in clinical practice where treatment decisions must rely on their practical relevance for the patient [26, 30, 31].

A recent systematic review [29] which analyzed 23 outcome studies in adolescent inpatient units, highlighted a critical gap: the lack of a clear understanding of clinical significance as distinct from statistical significance. While statistical significance determines whether observed differences are unlikely to be due to chance, it does not necessarily reflect the real-world impact of these changes on patients’ lives. Clinical significance, on the other hand, refers to meaningful and noticeable improvements in a patient’s mental health issues [32, 33]. Indeed, for both patients and clinicians, it is important to know whether individuals achieve a clinically significant improvement or, conversely, experience deterioration [34].

In brief, capturing clinically significant change is essential for understanding the heterogeneity and complexity of individual experiences, which statistical significance alone may overlook. Therefore, it has been emphasized that future research must extend beyond statistical significance to incorporate clinically meaningful changes, ensuring that observed improvements are not only statistically valid but also relevant in practice [29]. Although well-established methods for calculating individual changes derived from overall sample statistics are available and widely accepted [26] to our knowledge, no study has yet assessed the clinical significance of hospitalization outcomes in child and adolescent psychiatric units. Addressing this gap is essential to refining treatment approaches and optimizing care for young patients.

### Potential moderating factors related to clinical outcomes

Furthermore, there is considerable between-person variability in inpatient outcomes, and even the most effective treatments may not be efficient for all young people [35, 36]. Although many studies have tested sociodemographic (e.g., sex at birth, age), clinical (e.g., diagnosis) and intervention-related (e.g., medication, length of stay) moderators, their results remain contradictory [18, 22, 37–50]. This inconsistency likely reflects the substantial heterogeneity (e.g., models of care, admission policies or treatment programs) of inpatient psychiatric units (IPUs) which complicates cross-study comparisons and hampers the search for reliable moderators [51]. Moreover, most research links potential moderators to the severity of difficulties at discharge rather than to the changes during hospitalization, leaving the mechanisms that drive differential treatment response largely unexplored [29]. Clarifying not only who benefits, but also how improvements occur within specific IPUs is therefore essential for truly personalized and effective inpatient care.

### The current study

Therefore, this study seeks to (1) assess both statistically and clinically significant changes during hospitalization in an adolescent inpatient psychiatric unit, not only in overall mental health difficulties but also across specific domains, to gain a fine-grained understanding of its possible benefits; (2) identify moderating factors that may influence clinical changes (during hospitalization) as well as clinical outcomes (i.e., residual difficulties at discharge), thereby aligning with the principles of personalized medicine in adolescent psychiatric unit.

## Methods

### Procedure

Data were retrospectively extracted from medical records, specifically from each patient’s first hospitalization at the UHPA. Socio-demographic (e.g., age, sex), clinical (e.g., diagnosis) and intervention-related (e.g., medication, length of stay) variables were documented at admission and at discharge from inpatient stay. Moreover, mental health difficulties were measured using the Health of the Nation Outcome Scales for Children *[HoNOSCA; 52*,* 53*,* 54]*. The study was approved by the Cantonal Commission for Ethics in Human Research (CER-VD, #2021–02440) and has been conducted following the ethical standards laid down in the 1964 Declaration of Helsinki.

### Sample & setting

This retrospective study included 593 adolescents aged 13 to 18 years (M = 15.62, SD = 1.24) who were hospitalized for the first time (to avoid analytic bias from multiple readmissions) at the UHPA of the Lausanne University Hospital (CHUV) between 2014 and 2021. Sample’s characteristics are described in Table 2.

The UHPA at CHUV provides inpatient care for adolescents aged 13 to 18 years who present with acute psychiatric problems requiring hospitalization. The care is focused on managing the crisis and addressing the factors that likely contributed to the acute situation. The unit has a capacity of 10 patients and is supported by a multidisciplinary team, including doctors, nurses, social workers, psychologists, and dietitians. The care approach integrates various aspects of the patient’s life - educational, family, social, academic, and psychological. Treatment options may include (but are not limited to): individual treatment (e.g., psychiatric and psychotherapeutic care using psychopharmacological, psychodynamic and cognitive-behavioral brief treatment (including school and social aspects), occupational therapy, music therapy, group activities (e.g., reading, sports, outings) and family support (e.g., family interviews).

### Measures

#### Socio-demographic, clinical and intervention-related variables

The following information was routinely collected: sex at birth, age, occupation (i.e., employed, in training, on disability insurance or without occupation), provenance (i.e., home, institution, hospital or “*other*”), admission status (i.e., voluntary or placement for assistance), diagnosis according to the ICD-10 [World Health Organization, 55], medications (classified under antidepressants, antihistamines, antipsychotics, benzodiazepines, mood stabilizers, hypnotics and ADHD treatments), length of stay (i.e., duration of hospitalization, number of days between admission and discharge from hospital; M = 18.9 days; SD = 14.97; min 2 – max 62 days) and finally, the decision of discharge (i.e., by mutual agreement or on the initiative of the patient or caregiver, or “*other*”).

**Table 2:** Socio-demographic, clinical and intervention- related characteristics

Variables	Dimensions	*n*	%
Sex	Girls	365	61.6
	Boys	226	38.1
	*Missing*	2	0.3
Age^1^	Years	15.62	1.24
Occupation	Employed	8	1.3
	In training	478	80.6
	Without occupation	73	12.3
	Disability insurance	6	1
	*Missing*	28	4.7
Provenance	Home	459	77.4
	Institution	94	15.9
	Hospital	23	3.9
	Other	6	1
	*Missing*	11	1.9
Admission status	Voluntary	416	70.2
	Placement for assistance	155	26.1
	*Missing*	22	3.7
Diagnosis	F0x	7	1.2
	F1x	13	2.2
	F2x	44	7.4
	F3x	216	36.4
	F4x	189	31.9
	F5x	12	2
	F6x	20	3.4
	F7x	2	0.3
	F8x	14	2.4
	F9x	75	12.6
	*Missing*	8	1.4
Drugs	Antidepressants	124	20.9
	Antihistamines	197	33.2
	Antipsychotics	97	16.4
	Benzodiazepines	8	1.3
	Mood stabilizers	9	1.5
	Hypnotics	191	32.2
	ADHD treatment	5	0.8
	*Missing*	0	0
Decision to terminate	By mutual agreement	456	76.9
	Initiative of the caregiver	72	12.1
	Initiative of the patient	31	5.2
	Other	8	1.3
	*Missing*	26	4.5
Length of stay^1^	Admission - discharge interval (days)	18.9	14.97

^1^Data expressed in Mean and Standard Deviation (SD),* F0x * Somatoform disorders,* F1x * Use of psychoactive substances,* F2x * Schizophrenia spectrum disorders,* F3x * Mood (affective) disorders,* F4x * anxiety or obsessive-compulsive disorders,* F5x * Eating and sleeping disorders,* F6x * Personality disorders,* F7x* Intellectual disabilities,* F8x * Disorders of psychological development,* F9x* Behavioral and emotional disorders. *Missing*: no information

#### Mental health and psychosocial difficulties

The HoNOSCA measures psychopathological manifestations and psychosocial difficulties in adolescents [52, 56]. Its use in Child and Adolescent Mental Health (CAMHS) has been previously supported [56] and is required by the Swiss National Quality Association. Of note, the HoNOSCA is included in compulsory routine outcome monitoring in other countries, as well, for instance in Australia, Canada, Denmark, Germany, Italy, the Netherlands, Norway, Spain, and New Zealand [57].

More specifically, the HoNOSCA possesses good psychometric properties and can be considered appropriate for regular outcome monitoring (e.g., sensitivity to clinical changes), particularly in the evaluation of clinical change [58–60]. The HoNOSCA consists of 15 items, each rated (by trained clinicians) on a 5-point Likert scale ranging from 0 (“*no problems*”) to 4 (“*severe to very severe problems*”). In our study, we used only the initial 13 items, as they pertained directly to patient difficulties, ignoring the last two items (items 14 and 15), which did not have a direct link to the patients’ situation. Notably, the reliability of these final two items has been questioned and were left out in most previous studies using the HoNOSCA [28, 58]. The *“Total”* score (i.e., overall severity of physical, psychiatric and social problems associated with mental illness) is computed by summing the 13 items. However, relying solely on this composite score can be misleading; because each item addresses a different issue, significant decreases in item scores may indicate a significant clinical change, even if the *“Total”* score remains stable because of small changes in other items [27]. To address this ambiguity, we used two complementary approaches.

Following published recommendations, we applied a more advanced method to classify patients according to the severity of their overall problems [26, 61–63]. Patients were classified as; “*very severe*” if they had a score equal to or higher than 3 on at least two items (except for item 6 related to physical illness); “*moderately severe*” if they had a score equal to or higher than 3 on one item (except for item 6 related to physical illness); “*mild*” if they had at least one item with a score of 2; and “*subclinical*” if none of the scores was rated 2 or higher (see Table 2). Furthermore, to better capture domain-specific changes more sensitively, four subscores have also been computed in addition to the “*Total*” score. These subscores are supported by the original principal component analyses [63], namely *“Behavior”* (items 1–4), *“Impairment”* (items 5–6), *“Symptoms”* (items 7–9) and *“Social”* (items 10–13). It should be noted however that further studies did not replicate this structure [58, 64].

In addition to the use of classical measures to assess group-level significant differences, we computed reliable and clinically significant individual changes for all HoNOSCA scores via the Reliable Change Index [RCI; 33], a method widely recommended for identifying clinically meaningful change [e.g., 65]. In fact, it is a commonly recommended tool to precisely determine whether the observed shift in difficulties constitutes a real and genuine clinical change, thereby indicating a meaningful clinical evolution [66]. Furthermore, its use is supported by previous studies that have confirmed its application in various areas of psychology and has been recommended in a clinical context because it reliably tracks each patient’s progress, providing an opportunity to improve therapeutic approaches while ensuring that the treatments offered do not cause serious harm [66, 67].

In particular, to compute the RCI [33, 66], we used the following formula: $$ \:{\text{RCI}} = \frac{{{\text{X}}_{{\left( {{\text{Admission}}} \right)}} - \:{\text{X}}_{{\left( {{\text{Discharge}}} \right)}} }}{{{\text{S}}_{{{\text{diff}}}} }} $$. $$\:{\text{S}}_{\text{d}\text{i}\text{f}\text{f}}$$ was calculated as follows: $$\:{\text{S}}_{\text{d}\text{i}\text{f}\text{f}}=\:\sqrt{(2\times\:\:({\text{S}\text{E}}^{2})}$$ where $$ {\text{SE }} $$$$ = {\text{SD}}\left( {\sum \: {\text{X}}_{{\left( {{\text{Admission}}} \right)}} } \right) $$$$ \times \:\:\sqrt {(1 - {\text{test}} - {\text{retest}}\:{\text{reliability}})} $$. We used the test–retest reliability index (i.e., the intraclass correlation coefficients (ICCs) reported by Boon, de Boer [26] for each HoNOSCA score (“*Total*”, “*Behavior*”, “*Impairment*”, “*Symptoms*” and “*Social*”). We also categorized the RCI as “*Improved*” if over 1.96, as “*Deteriorated*” if below − 1.96 and as “*No reliable change*” if between − 1.96 and 1.96 [33] .

### Data analyses

As data followed a Gaussian distribution, parametric tests were used. Statistical differences were assessed with the use of Student t-test for paired samples (comparing admission to discharge scores). To examine moderating factors of hospitalization, preliminary univariate analyses were conducted to assess associations (i.e., Bravais-Pearson correlation coefficients) between continuous RCI scores or raw HoNOSCA scores at discharge and socio-demographic, clinical, and intervention-related variables (see Supplementary Material, Table S1). Subsequently, multivariate linear regression analyses were performed, using socio-demographic, clinical, intervention-related variables as predictors and either RCI scores (used in their continuous form, assessing clinical changes during hospitalization) or raw HoNOSCA scores at discharge (assessing clinical outcomes) as outcome measures. To enhance the robustness of our model and ensure that associations with clinical change or discharge outcomes were not solely driven by baseline conditions, we included the “*admission Total score*” to control for initial severity. All tests were performed with SPSS v.27 and significance has been set at *p* < .05.

## Results

### Descriptive results

Table 3 presents the distribution of patients across the four severity categories (i.e., “*very severe*”; “*moderately severe*”; “*mild*” and “*subclinical*”) at admission and discharge, based on the classification of the total HoNOSCA score. At both time points, the largest proportion of patients fell into the “*very severe*” category (about two-thirds of the sample). In contrast, the “moderately severe” and “mild” categories each accounted for around 15–18% of patients, while the “*subclinical*” category included only a very small proportion (around 2%).

**Table 3:** Prevalence of the severity categories

	Admin		Discharge	
	n	%	n	%
Subclinical	8	1.35	16	2.70
Mild	85	14.33	89	15.01
Moderately severe	86	14.50	105	17.71
Very severe	414	69.81	383	64.59

Patients are classified as: “very severe” if they have a score equal or higher of 3 on at least two HoNOSCA’s items (item 6, physical illness, excluded); “moderately severe” if they have a score equal or higher of 3 on one items (item 6, physical illness, excluded); “mild” if they have at least one item scoring of 2 and “subclinical” if they were not rated with scores of 2 or higher

### Statistical changes

Table 4 summarizes the mean HoNOSCA total score and subscales at admission and discharge, as well as paired t-tests and effect sizes (Cohen’s d). Results indicated significant differences between admissions and discharge for the “*Total*” score, as well as for the “*Behavior*” and “*Symptoms*” subscales. No significant differences were found for the “*Impairment*” and “*Social*” subscales.

**Table 4:** Descriptive data and comparison of admission vs. discharge HoNOSCA scores

	Admission		Discharge				
	M	SD	M	SD	*t*	*p*	Cohen’s d
Total	16.44	7.92	15.47	7.59	2.41	0.016	9.81
Behavior	4.24	3.35	3.75	3.28	2.87	0.004	4.17
Impairment	1.56	1.69	1.51	1.61	1.27	0.205	1.13
Symptoms	4.28	2.28	4.03	2.18	2.15	0.032	2.77
Social	6.36	3.35	6.18	3.23	1.17	0.241	3.64

*M* Mean,* SD* Standard deviation

### Clinical changes

**Fig. 1:**
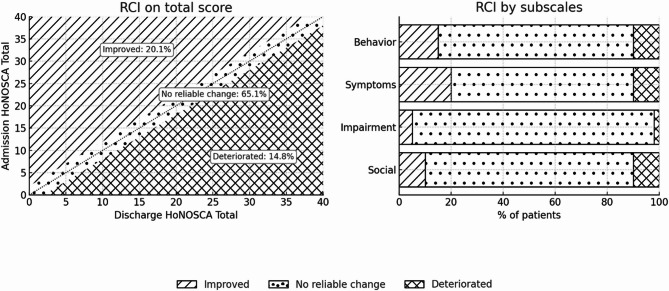
*Clinical change (RCI categories) from admission to discharge in HoNOSCA scores*:

Figure 1 illustrates the distribution of patients across the RCI categories. On the ”*Total*” score, most patients fell within the “*no reliable change*” category, with smaller proportions in the “*improvement*” and “*deterioration*” categories. When considering the four subscales, the proportion of improved patients ranged from 2.7% for « *Impairment »*, 10.1% for « *Social »*, 17.0% for « *Behavior »*, to 19.1% for « *Symptoms »*. Deterioration was less frequent, with percentages of 2.2% for « *Impairment »*, 9.4% for « *Social »*, 10.8% for « *Behavior »*, and 13.7% for « *Symptoms »*. In each subscale, most patients (67.3%–95.1%) remained in the no reliable change category.

### Multivariate analyses

Multivariate regression analyses conducted on RCI scores did not reach significance, indicating that the models were not able to explain a significant part of variance in RCI scores (i.e., clinical changes during hospitalization). In contrast, all regression models explained a significant part of the variance of discharge scores (i.e., residual difficulties, Table 5). Indeed, the analyses of variance were significant for the *“Total”* score (*F*(13, 475) = 5.55, *p* < .001, *R*^*2*^ = 0.132), *“Behavior”* (*F*(13, 475) = 4.35, *p* < .001, *R*^*2*^ = 0.106), *“Impairment”* (*F*(13, 475) = 7,66, *p* < .001, *R*^*2*^ = 0.173), *“Symptoms”* (*F*(13, 475) = 3.46, *p* < .001, *R*^*2*^ = 0.087) and *“Social”* (*F*(13, 475) = 5.25, *p* < .001, *R*^*2*^ = 0.126) scores.

**Table 5:** Results of regression analyses on discharge scores HoNOSCA scores

	Total		Behavior		Impairment		Symptoms		Social	
	β	*p*	β	*p*	β	*p*	β	*p*	β	*p*
Age	-0.05	0.262	-0.02	0.645	-0.07	0.114	0.01	0.713	-0.07	0.102
Sex (girls = 1)	-0.02	0.590	0.04	0.334	**-0.14**	**0.002**	0.04	0.325	-0.06	0.166
Lenght of stay	-0.03	0.497	**-0.11**	**0.021**	-0.00	0.910	0.01	0.817	0.03	0.526
F3x	-0.07	0.272	-0.05	0.422	-0.17	0.005	-0.06	0.336	0.01	0.787
F4x	-0.01	0.880	0.02	0.722	-0.11	0.102	-0.04	0.582	0.03	0.642
F9x	-0.03	0.597	0.09	0.138	-0.10	0.082	**-0.14**	**0.015**	-0.01	0.856
Antipsychotic	**0.13**	**0.011**	**0.17**	**0.001**	0.07	0.153	**0.12**	**0.019**	0.01	0.752
Antidepressor	0.03	0.458	0.00	0.996	0.00	0.870	0.02	0.680	0.06	0.176
Mood	0.07	0.120	0.07	0.122	0.00	0.879	0.02	0.709	0.07	0.082
Voluntary admin	-0.09	0.066	**-0.10**	**0.036**	0.031	0.495	-0.03	0.607	**-0.10**	**0.031**
Occupation	**-0.17**	**< 0.001**	**-0.09**	**0.045**	**-0.18**	**< 0.001**	-0.00	0.912	**-0.20**	**< 0.001**
Agreement for discharge	**-0.15**	**0.001**	**-0.14**	**0.002**	0.00	0.973	**-0.23**	**< 0.001**	-0.04	0.264
Admission total score	0.12	0.007	0.00	0.975	**0.23**	**< 0.001**	0.01	0.838	**0.17**	**< 0.001**
*R* ^*2*^	0.118		0.106		0.128		0.086		0.101	

Bold: significant association

In particular, several socio-demographic, clinical and intervention-related factors were associated with discharge scores. Being male was related to higher “*Impairment*” score, while having an occupation before hospitalization was linked to lower “*Total*”, “*Behavior*”, “*Impairment*” and “*Social*” scores at discharge. Voluntary admission was associated with lower “*Behavior*” and “*Social*” scores, and a diagnosis of “Behavioral and emotional disorder (F9.x)” to lower “*Symptoms*” scores at discharge. A longer length of stay was linked to lower “*Behavior*” scores at discharge, the use of antipsychotic medication to higher “*Total*”, “*Behavior*” and “*Symptoms*” scores, and finally the presence of an agreement between patients and clinician regarding discharge to lower “*Total*”, “*Behavior*” and “*Symptoms*” scores at discharge. Higher admission scores were also controlled for in the models and were significantly associated with higher “*Impairment*” and “*Social”* scores at discharge.

## Discussion

The current study aimed to assess statistically and clinically significant changes related to hospitalization in an IPU, and to investigate potential moderating factors that may be associated with clinical changes during hospitalization and outcomes at discharge. The study identified statistically significant reductions in certain HoNOSCA scores from admission to discharge, indicating a relative decrease in the severity of difficulties; a robust group-level effect that translated into clinically meaningful changes for only some of the adolescents. Indeed, while a portion of patients showed individual clinical improvement, the majority remained stable, with a mild percentage experiencing deterioration. While clinical changes during hospitalization remained largely unexplained, factors such as sex at birth, the presence of behavioral and emotional disorders (F9.x), antipsychotic use, voluntary admission, pre-hospitalization occupation, patient-clinician discharge agreement, and length of stay accounted for a significant proportion of various clinical outcomes at discharge.

### Severity of difficulties

Our results indicate that a high proportion of patients were classified as *" very severe" * at both admission (70%) and discharge (65%), likely reflecting the crisis IPU’s role in managing acute and severe cases. To our knowledge, only two other studies, have used this specific HoNOS-based severity classification. One study using data from ten CAMHS in the Netherlands reported a decrease in *“very severe*” cases from 50.6% at admission to 4.7% at discharge [26], whereas another reported a 22% rate in a single-time-point ambulatory adult sample [61]. These differences underscore the persistence of a severe status in our sample, which may be attributable to the IPU’s emphasis on short-term stabilization rather than complete symptom remission, with many patients generally transitioning to other services [39].

### Statistical changes

Nevertheless, alongside these descriptive findings, our statistical results revealed a significant reduction in overall difficulties (operationalized as the “*Total*” score) during hospitalization, consistent with previous studies [28, 29, 37, 68] and suggesting inpatient care as a supportive intervention for most adolescents [22].

Notably, differences were most pronounced in the “*Behavior*” and “*Symptoms*” domains, whereas “*Impairment*” and “*Social*” remained statistically unchanged, indicating that hospitalization primarily targets specific clinical issues, requiring immediate intervention. In particular, it effectively reduced difficulties such as suicidal thoughts and behaviors (i.e., self-harm), anxiety, depressive thoughts, delusions, and agitation - issues often prioritized during hospitalization [69]. In contrast, socio-functional issues, such as academic performance, disabilities, and interpersonal relationship problems, are best addressed in the long term through outpatient care [16]. In this regard, well-planned discharge strategies have been shown to strengthen the continuity of care and enhance the overall effectiveness of hospitalization [18].

### Clinical changes

*Reliable* and *clinical* improvement in the “*Total*” score was achieved in 20% of the patients, while 65% of the patients remained stable, and 14% experienced deterioration. This finding is partly consistent with a study conducted in the Netherlands in 10 long-term IPUs, who observed that most adolescents remained stable with roughly one-fifth showing improvement when using the RCI, although our higher deterioration rate (14% vs. 2.9%) likely reflects our shorter average stay (19 days in our institution versus 273 days in their CAMHS) [26]. Although shorter inpatient stays can address immediate crises, they may not suffice for more complex or chronic cases, where statistically significant gains may remain too modest to achieve clinical significance. This limitation could partly account for the deterioration rate in our study, alongside multiple interrelated factors such as transient side effects from necessary medication adjustments, the abrupt confrontation with personal difficulties and the challenges of establishing a therapeutic alliance - often complicated by adolescents’ pursuit of autonomy and tendency toward opposition [70–73].

Besides, our domain-specific analyses indicate that the rate of clinical improvement can vary depending on the dimensions assessed (2.7% for “*Impairment*” RCI scores, up to 19.1% for “*Symptoms*” RCI scores). As with statistical significance, this variation suggests that acute clinical issues may respond better to short-term inpatient care than broader socio-functional difficulties, which may require specialized interventions outside the scope of emergency psychiatric care [16]. Therefore, future studies should examine how a more comprehensive treatment plan, integrating both acute and long-term care approaches, can better address such needs within the broader continuum of mental health care [18, 74].

In summary, our findings are in line with Hsu’s meta-analysis [29], which showed that adolescent IPUs are statistically effective in improving outcomes. While the clinical significance is modest, these results are promising given the primary goals of care, yet warrant further investigation as some patients deteriorated. Moreover, the distinction between statistical and clinical significance remains crucial; it is entirely possible to observe statistically significant group-level effects even when most patients do not reach the threshold for individually meaningful improvements. In this context, researchers and clinicians need effective methods to monitor clinical patient progress, and our study highlights the RCI’s value in detecting clinically significant improvements or deterioration to guide care [34, 61].

### Moderating factors

In our study, various socio-demographic, clinical, and intervention-related factors were significantly associated with discharge outcomes, highlighting for whom treatment may be more effective - yet providing no insight into the underlying processes by which patients actually improve (or fail to improve) during the inpatient stay.

#### Socio-demographic factors

First, being male (sex assigned at birth) was linked with greater “*impairment*” problems at discharge. Although boys often experience more severe outcomes than girls [75], this difference may stem partly from delayed recognition and articulation of difficulties; in contrast, girls tend to seek help sooner, facilitating earlier, more targeted interventions [76]. Additionally, most adolescents who engaged in occupational activities before admission experienced fewer mental health issues at discharge (except symptomatic problems), reinforcing the idea that structured activities provide continuity, purpose, and social connection, ultimately easing their preparation to transition from inpatient care to everyday life [77, 78].

#### Clinical factors

Besides, voluntary admission emerged as an important clinical factor, as it was associated with lower severity in both “*behavioral”* and “*social”* difficulties at discharge. This finding is consistent with previous one [48, 79], suggesting that voluntary admission facilitates the establishment of a therapeutic relationship by actively engaging the adolescent in the therapeutic process from the outset, thereby contributing to better outcomes. Beyond the mode of admission, individual diagnostic profiles also appeared relevant : patients diagnosed with “behavioral and emotional disorders” (F9.x) showed less severity of their “*symptoms*” (i.e., emotional and psychotic symptoms) at discharge. Still, high comorbidity in adolescent inpatient settings complicates distinct diagnostic categorization and treatment responses [18].

#### Intervention-related factors

Longer hospital stays coincided with less “*behavioral”* issues at discharge, likely because clinicians had more time for in-depth assessments - sometimes involving medico-legal - and could provide respite from a destabilizing external environment [51]. In an era of rising demand for adolescent psychiatric care, adjusting the length of stay is one of the few levers clinicians can use to balance finite resources with patient needs [80]. Yet the notion of an “*ideal*” length stay must accommodate clinical realities such as optimizing medication regimens and mitigating side effects, given that antipsychotics - often essential for stabilizing acute symptoms - are frequently associated with dose-dependent adverse effects, which, in this study, were particularly linked to more “*behavioral”* and “s*ymptomatic”* problems at discharge [81, 82]. In fact, determining the optimal pharmacological dose is challenging, as therapeutic effects can be delayed and vary between individuals, so the initial dosage and its early outcomes are seldom optimal [83]. Rapid medication titration helps reduce hospital stay and costs but requires careful monitoring to balance efficacy and side-effects, ensuring adherence and preventing relapses [84]. Conversely, planned and consensual discharges, jointly agreed by the care team and the patient, were protective not only in these *behavioral* and *symptomatic* domains but also in overall difficulties, presumably by fostering a robust therapeutic alliance and smoother transition to outpatient clinics and daily life after hospitalization [48, 50, 85–87].

Collectively, these findings underscore that integrating individual backgrounds with the inpatient experience central to optimizing hospitalization outcomes, with socio-demographic, clinical, and intervention-related factors informing which patients might benefit most - though they do not fully explain how or why changes occur [73].

### Clinical implications

Anchored in the stepped-care model (see Table 1 above), which reserves hospitalization for acute or severe psychiatric issues [15], our findings emphasize the need for interventions that address the multifaceted nature of adolescents’ difficulties and the collaboration with the outpatient facilities. Although short-term inpatient care effectively stabilizes acute symptoms or behavioral crises, issues related to impairment and social functioning often persist. Table 6 highlights a domain-specific pattern, underlining the importance of identifying which types of patients’ difficulties and characteristics benefit most from high-intensity inpatient interventions and which require complementary or alternative approaches. Within the stepped-care framework, such insight enables the early identification of clinical priorities and helps orient adolescents toward the most appropriate care trajectory. In turn, personalized planning may improve clinical efficiency and reduces the emotional burden often experienced by patients and families during hospitalization. Moreover, when extended hospital stays are necessary, early coordination with community-based services, rehabilitation programs, and outpatient specialists becomes essential to ensure continuity of care. On a broader scale, at a health care system level, recognizing the variability in therapeutic needs allows for more strategic resource allocation, ensuring that specialized support is concentrated where it is most needed [88].

**Table 6:** Clinical implications

Domains of difficulties (HONOSCA’s scores)	Observed Effect of Hospitalization	Associated Moderators (+: more; –: less severity at discharge)
*Overall* (Total HoNOSCA)	Significant statistical; some clinical Improvement	+ Antipsychotics – Occupation before hospitalization – Agreement on discharge
*Behaviors* (disruptive behavior, agitation, hyperactivity, self-harm, alcohol-drugs addiction)	Significant statistical; some clinical Improvement	+ Antipsychotics – Voluntary admission – Longer length of stay – Agreement on discharge – Occupation before hospitalization
*Symptoms* (hallucinations, delusions, abnormal perceptions, anxiety, depression, phobias, obsessions/compulsions, eating disorders)	Significant statistical; some clinical Improvement	+ Antipsychotics – F9.x diagnosis – Agreement on discharge
*Impairment* (academic or language skills, problems related to a disability (motility))	Minimal (stable, no significant change)	+ Being a male – Occupation before hospitalization
*Social* (social interactions, daily living activities, self-care (eating, bathing, dressing)	Minimal (stable, no significant change)	– Voluntary admission – Occupation before hospitalization

### Strengths and limitations

Among its strengths, this study relied on valid and reliable measures (HoNOSCA and the RCI) to assess short-term interventions, aligning with evidence-based medical practice [89]. Moreover, its naturalistic approach in a real-world crisis context enhances generalizability to similar settings [90], and a large sample of nearly 600 patients, along with a thorough examination of multiple factors, strengthens its design and statistical power, ultimately supporting the reliability of the findings and the identification of several clinically significant outcomes [91].

At the same time, some limitations must be acknowledged and temper the conclusions. Although HoNOSCA ratings followed standardized procedures, assessments may have been influenced by inter-rater variability, as admission and discharge evaluations were not always carried out by the same clinicians [22]. Moreover, the exclusive reliance on clinicians ratings without incorporating direct input from patients and families restricts the validity of the findings about the subjective experience of changes in difficulties intensity [92]. Additionally, the retrospective nature of the design also imposes important constraints: information was drawn from chart data, which reduces temporal precision and prevents a clear understanding of when difficulties emerged, at what point specific interventions, such as medication adjustments, were implemented, reducing the ability to capture the dynamic nature of mental health issues evolution during hospitalization [93, 94]. This lack of temporal resolution also limits the ability to understand how different clinical variables may evolve and interact during hospitalization. Equally important, the absence of a control group makes it impossible to determine to what extent observed changes can be attributed to hospitalization itself, rather than to spontaneous fluctuations, natural regression to the mean, or concurrent treatments [46, 95]. Finally, the study focused mainly on relatively static moderators, such as diagnosis or admission status [96, 97]. Yet certain difficulties observed in adolescents, including suicidal ideation and behaviors, are highly dynamic and can fluctuate over short periods. To better understand how such difficulties evolve during hospitalization, it would be necessary to examine more proximal moderators, (e.g., emotional regulation, or family support) that may co-vary with fluctuations in mental health difficulties and may be crucial in shaping short-term trajectories but could not be captured in the present study [98]. Future prospective studies should address these challenges.

## Conclusion

In conclusion, this study highlights a mixed pattern in adolescent psychiatric hospitalization: while statistically significant reductions in behavioral and symptomatic difficulties were observed, clinically meaningful improvements were limited. Indeed, hospitalization appears to provide short-term stabilization for most patients, without necessarily leading to meaningful clinical improvement, while a small but important subset experienced deterioration, highlighting the need for closer monitoring and more focused support. Several moderating factors, such as sex assigned at birth, diagnosis, antipsychotic use, voluntary admission, pre-hospitalization occupation, therapeutic agreement at discharge, and length of stay, were associated with discharge outcomes, suggesting opportunities to better align care pathways with patient characteristics. Overall, these findings may support the role of inpatient care as a time-limited intervention within a broader continuum of services to address more persistent and complex difficulties. Finally, future research should also explore more dynamic, proximal moderators of change and incorporate patient perspectives to inform more responsive, effective and personalized care strategies.

## Supplementary Information

Below is the link to the electronic supplementary material.

Supplementary Material 1.

## Data Availability

No datasets were generated or analysed during the current study.
