# Supplementary material for: Adolescent psychiatric hospitalization: a naturalistic observational study of statistical and clinical outcomes and moderating factors
Source: Child Adolesc Psychiatry Ment Health. 2025 Oct 28;19:117. doi: 10.1186/s13034-025-00972-8 (PMC12570707; doi:10.1186/s13034-025-00972-8)
Supplement: Supplementary file 1 — Supplementary Material 1. [file 13034_2025_972_MOESM1_ESM.docx]

**Supplementary Material**

**Table S1.** Univariate associations between reliable change indexes (RCI), discharge scores and socio-demographic and clinically variables.

|  | RCI | | | | | Discharge | | | | | | |
| --- | --- | --- | --- | --- | --- | --- | --- | --- | --- | --- | --- | --- |
|  | Total | Behavior | Impairment | Symptoms | Social | Total | Behavior | Impairment | Symptoms | | Social | |
| Age | .05 | .06 | .00 | .05 | .04 | .02 | .01 | .02 | | .06 | | -.01 |
| Lenght of stay | .08 | .06 | .00 | .09^*^ | .07 | .01 | -.09^*^ | .07 | | .05 | | .05 |
| Sex (girls = 1) | -.06 | -.06 | .00 | -.06 | -.05 | -.10^*^ | -.02 | -.24^**^ | | .03 | | -.12^**^ |
| F3x | .05 | .02 | .01 | .02 | .08 | -.06 | -.06 | -.12^**^ | | .02 | | -.04 |
| F4x | -.09^*^ | -.07 | -.03 | -.11^*^ | -.06 | -.05 | -.02 | -.05 | | -.01 | | -.05 |
| F9x | -.01 | -.02 | .03 | .01 | -.02 | .03 | .11^**^ | -.01 | | -.10^*^ | | .03 |
| Antipsychotic | .05 | .04 | .01 | .08 | .03 | .18^**^ | .13^**^ | .20^**^ | | .13^**^ | | .10^*^ |
| Antidepressor | -.04 | -.03 | -.03 | -.05 | -.02 | .02 | .03 | -.03 | | .06 | | .00 |
| Mood | -.03 | -.06 | .03 | -.05 | .01 | .07 | .05 | .04 | | .04 | | .07 |
| Voluntary admin | -.09^*^ | -.10^*^ | -.04 | -.09^*^ | -.04 | -.14^**^ | -.11^**^ | -.10^*^ | | -.01 | | -.16^**^ |
| Occupation | .03 | .05 | .03 | .00 | .02 | -.21^**^ | -.16^**^ | -.21^**^ | | -.01 | | -.23^**^ |
| Agreement for discharge | .09^*^ | .08^*^ | .05 | .05 | .09^*^ | -.11^**^ | -.11^**^ | .01 | | -.18^**^ | | -.04 |

*Note.* Bravais-Pearson coefficients of correlation for continuous variables or ANOVA chi-square for categorial variables. * *p* <.05; ** *p* <.01; *** *p* <.001.
